# Cynomolgus monkeys (*Macaca fascicularis*) experimentally infected with B19V and hepatitis A virus: no evidence of the co-infection as a cause of acute liver failure

**DOI:** 10.1590/0074-02760160013

**Published:** 2016-04

**Authors:** Luciane Almeida Amado Leon, Renato Sergio Marchevsky, Ana Maria Coimbra Gaspar, Rita de Cassia Nasser Cubel Garcia, Adilson José de Almeida, Marcelo Pelajo-Machado, Tatiana Xavier de Castro, Jussara Pereira do Nascimento, Kevin E Brown, Marcelo Alves Pinto

**Affiliations:** 1 Fundação Oswaldo Cruz, Instituto Oswaldo Cruz, Laboratório de Desenvolvimento Tecnológico em Virologia, Rio de Janeiro, RJ, Brasil; 2 Fundação Oswaldo Cruz, Bio-Manguinhos, Laboratório de Controle de Neurovirulência, Rio de Janeiro, RJ, Brasil; 3 Universidade Federal Fluminense, Instituto Biomédico, Departamento de Microbiologia e Parasitologia, Niterói, RJ, Brasil; 4 Universidade Federal do Estado do Rio de Janeiro, Escola de Medicina e Cirurgia, Hospital Universitário Gaffrée e Guinle, Unidade de Hematologia, Rio de Janeiro, RJ, Brasil; 5 Fundação Oswaldo Cruz, Instituto Oswaldo Cruz, Laboratório de Patologia, Rio de Janeiro, RJ, Brasil; 6 Health Protection Agency, Virus Reference Department, London, UK

**Keywords:** parvovirus B19, hepatitis A, acute liver failure, co-infection, cynomolgus monkeys

## Abstract

This study was conducted to analyse the course and the outcome of the liver disease in the co-infected animals in order to evaluate a possible synergic effect of human parvovirus B19 (B19V) and hepatitis A virus (HAV) co-infection. Nine adult cynomolgus monkeys were inoculated with serum obtained from a fatal case of B19V infection and/or a faecal suspension of acute HAV. The presence of specific antibodies to HAV and B19V, liver enzyme levels, viraemia, haematological changes, and necroinflammatory liver lesions were used for monitoring the infections. Seroconversion was confirmed in all infected groups. A similar pattern of B19V infection to human disease was observed, which was characterised by high and persistent viraemia in association with reticulocytopenia and mild to moderate anaemia during the period of investigation (59 days). Additionally, the intranuclear inclusion bodies were observed in pro-erythroblast cell from an infected cynomolgus and B19V Ag in hepatocytes. The erythroid hypoplasia and decrease in lymphocyte counts were more evident in the co-infected group. The present results demonstrated, for the first time, the susceptibility of cynomolgus to B19V infection, but it did not show a worsening of liver histopathology in the co-infected group.

Parvovirus B19 (B19V), the agent that causes erythema infectiosum (fifth disease), infects the erythroid progenitor cells, causes maturation arrest in the erythroid series, and bone marrow (BM) failure in immunocompromised patients. Infections caused by B19V and other primate erythroviruses are known to be strongly influenced by the immunologic and haematologic status of hosts. In general, healthy immunocompetent adults show acute infection, marked with a temporary depression of erythropoiesis. The appearance of specific antibodies in blood may be accompanied by polyarthritis, arthralgia, myocarditis, and immune complex deposition at tissues, conferring an immune-mediated nature to the disease (Anderson et al. 1985).

An increasing spectrum of clinical manifestation of B19V infection has been described (Bathla et al. 2014), including hepatitis, which is commonly caused by hepatotropic viruses (A-E) (Hatakka et al. 2011, Rauff et al. 2011, Huang et al. 2012). On the basis of its DNA detection in the liver of patients with acute liver failure (ALF) associated with BM aplasia and in the serum of patients with ALF of unknown aetiology, B19V has been implicated as an aetiological agent for ALF-associated aplastic anaemia (Aoyagi et al. 1987, Langnas et al. 1995, Bernuau et al. 1999, Abe et al. 2007, Dwivedi et al. 2009, Huang et al. 2012, Bathla et al. 2014).

ALF is a severe complication of acute viral hepatitis, which occurs in less than 1% of the cases and is generally caused by hepatitis A-E viruses either single or in combinations (Dwivedi et al. 2009). However, previous reports have demonstrated a hepatic severity significantly greater in patients with hepatotropic viruses (A and E) co-infected with B19V (Ozcay et al. 2006, Kishore & Sen 2009). Recently, one study carried out with 48 paediatric patients with ALF showed the presence of the B19V genome in 19 (39%) cases, of which 13 (27%) were also positive for IgM antibodies against other hepatitis viruses (Dwivedi et al. 2009). Comparing the clinical characteristics and outcomes of patients having liver failure associated with B19V alone and co-infected with hepatitis A, B, C or E, the results showed that the disease was significantly more severe in patients with B19V co-infection.

Although fulminant hepatitis A infection is rare (Jeong & Lee 2010), it is a frequent cause of ALF among children (Aydoğdu et al. 2003, Jayakumar et al. 2013). Patients with fulminant hepatitis A are known to have a spontaneous better prognosis than those induced by other aetiology (Ozcay et al. 2006). However, poor prognosis of the fulminant hepatitis A patients has been related to B19V co-infection (Chehal et al. 2002, Ozcay et al. 2006). In addition, a fatal case of a child with ALF due to infections with hepatitis viruses A and E together with B19V was reported (Kishore & Sen 2009). There are, however, many conflicting results about the association of B19V with other viral infections inducing ALF (Wong et al. 2003, Kumar et al. 2006, Opaleye et al. 2011) or other worst out comes (Mogensen et al. 2010). Thus, many aspects of the role of co-infection in the outcome of the hepatic disease remain unclear.

Therefore, we conducted an experimental infection study to analyse the course and the outcome of the liver disease in the B19V/HAV co-infected animals in order to evaluate a possible synergic effect of the co-infection with respect to hepatic injury. We also investigated the susceptibility of cynomolgus monkey to B19V by haematological and virological parameter, in order to determine if this animal may be a useful model for further new studies about B19V infection.

## MATERIALS AND METHODS

*Animals* - This study was carried out in strict accordance with the recommendations of national and international guidelines for the care and use of laboratory animals. This specific experimental protocol was reviewed and approved by the Oswaldo Cruz Foundation (Fiocruz) (Rio de Janeiro, Brazil) Ethical Committee for the Use of Animals (resolution P0064-00). All surgery was performed under anaesthesia and all efforts were made to minimise suffering.

Nine clinically healthy, young adult (weighing 3-5 kg) cynomolgus monkeys, ranging in age from three-four years old, from the Department of Primatology, Institute of Science and Technology in Biomodels (Fiocruz), were used and confirmed to be seronegative for specific anti-HAV and anti-B19V immunoglobulins by a commercial immunoassays. All animals have health certificates, which guarantee the absence of infectious diseases. A serological survey confirmed that they were free of simian immunodeficiency virus and simian type D retrovirus. During the study and quarantine periods, the monkeys were housed individually, in order to prevent cross infection among inoculated and noninoculated cynomolgus monkeys, in stainless-steel squeeze-back cages in a climate-controlled room (temperature 21 ± 1ºC and relative humidity 55 ± 5%) with a 12 h light/dark cycle. They were fed daily with a commercially available primate diet supplemented with fresh fruits and vegetables. Water was provided *ad libitum*. The cage used in our study to house each monkey was 0.77 m (height) x 0.60 m (width) x 0.68 m (depth) (Suburban Surgical CO, Inc, USA). In order to minimise the stress of the animals throughout the study period, they were subjected to an environmental enrichment program consisting of a series of measures that modify the physical and social aspects, improving the quality of life of animals, such as: (i) stainless steel mirror - made of polished stainless steel attached to the front cage grid, allowing the animal to move it to explore the environment; (ii) foraging tray - made of stainless steel containing a plastic carpet with recesses attached to the cage, allowing the animal to handle some items (i.e., cereal bar fragments, raisins, sunflower seeds) arranged on the tray; (iii) PVC of biting - made with PVC pipe with perforations along its length and threaded caps at the ends, which allow the placement of items (i.e., cereal bar pieces, raisins, rice grains); (iv) electronic equipment offering classical music in CD’s; (v) microwave popcorn - popcorn offer for at least one day of the week; (vi) medicinal herbs - offering various medicinal herbs in at least one week day (mint, lemon balm, chamomile, and calendula). The animals were in good physical health with normal baseline biochemical and haematological values.

*Inocula* - The B19V inoculum was obtained from the serum of a 68-year-old male Afro-Brazilian patient (anti-HAV IgG negative) diagnosed as having sickle cell disease, showing unresponsive anaemia and thrombocytopenia. The BM biopsy confirmed myelodysplasia and inclusions similar to parvovirus (Rio de Janeiro B19V outbreak occurred in 2004-2006). To B19V DNA detection and genotyping a seminested polymerase chain reaction (PCR) was performed using primers (P12F/P16R and P13F/P16R) that amplify a partial VP1/VP2 region of the B19V genome (Durigon et al. 1993). The 476-bp fragment was purified using a PCR purification kit (QIAquick^®^ DNA Mini Kit; Qiagen, UK) and subjected to direct sequencing in both directions using the Big Dye Terminator Cycle Sequencing Kit on a 3130 Genetic Analyzer (Applied Biosystems, USA). Sequences were aligned using BioEdit Sequence Alignment Editor v.7.0.5.2 (Ibis Biosciences, USA) and were compared with other sequences available in GenBank. Sequence analysis characterised this fragment as genotype 1a (GenBank accession KU342655). The viral load (VL) in the patient serum was 10^5^ copies/mL. Each animal received 1 mL of this serum *via* the intravenous route.

The HAV strain HAF-203 (GenBank accession AF268396) was isolated from stools of a Brazilian child with sporadic hepatitis A collected as part of previously published research (Gaspar et al. 1992). The stool samples were diluted 1% (w/v) in phosphate-buffered saline (10 nM sodium phosphate, 0.15 M NaCl) with penicillin (100 IU⁄mL) and streptomycin (100 mg⁄mL), clarified by low-speed centrifugation, and filtered through a 0.45 mm membrane. This inoculum was quantified by real-time PCR (RT-PCR) (3 x 10^5^ copies/mL).

*Experimental design* - The study was designed to evaluate clinical and laboratory findings of three groups of cynomolgus: (i) three animals with B19V inoculum only *(b*-U5, *b*-T9, *b*-D2); (ii) three animals co-infected with B19V and HAV (*bh*-T15,*bh*-G7, *bh*-Q14); (iii) three animals infected with HAV alone (*h*-V3, *h*-V13 and*h*-H8). Pre-inoculation serum, BM, and liver biopsies were collected prior to inoculation to establish individual baseline values. Clinical features and rectal temperatures were checked daily as well as collection of faeces. All invasive procedures were performed under ketamine hydrochloride (20 mg/kg) (Vetanarcol; König, Argentina) and xylazine (0.1 mg/Kg) (Syntec Do Brasil, Brazil), anaesthesia, administered intramuscularly, and all efforts were made to minimise suffering. All animals were monitored for 59 days; the periodicity of surgical procedures and blood collection were as follows: zero, seven, 14, 19, 31, 45, and 59 days post-infection (dpi). Liver biopsy samples were taken by Menghini needle puncture (16G) under the right side floating rib once a month guided by ultrasonography. BM was approached by aspiration puncture (14G) of the right side iliac crest for morphological analysis of red cell linage. The after surgical procedures were well tolerated by all cynomolgus monkeys, they were clinically followed twice-a-day, any additional analgesic procedure was necessary during this study, and surgery was healed two days after puncture. All monkeys were euthanised by total exsanguination under deep anaesthesia with ketamine/xylazine and necropsy was performed. The monkeys inoculated with B19V were euthanised at the end of the follow up (59 dpi), while the monkeys monoinoculated only with HAV were euthanised on days 30 (*h*-H8), 45 (*h*-V13), and 59 (*h*-V3) dpi. After necropsy, liver fragment samples were excised for histological study.

*HAV and B19V antibodies detection* - Serum samples were assayed for detection of total anti-HAV antibodies using a commercial kit (Bioelisa HAV 96T Kit; Biokit SA, Spain) according to the manufacturer’s instructions. IgG anti-B19V antibodies were detected using a commercial immune enzymatic assay (Biotrin International Ltd, Ireland). All serological tests were performed according to manufacturer’s instruction with minor modifications. These tests were based on cross reactivity between human and cynomolgus IgG antibodies (de Swart et al. 1998, Clark et al. 2013). The cut-off points for HAV and B19V antibodies detection were evaluated using receiver operating characteristic (ROC) curve analysis (MedCalc, Belgium). The ability of the model to differentiate between positive and negative monkeys for total anti-HAV or IgG anti-B19V (discrimination) was quantified using the area under the curve (AUROC) test (Hanley & McNeil 1982, Steyerberg et al. 2001).

*Liver enzymes* - Serum samples collected from the monkeys were analysed for alanine aminotransferase (ALT) and aspartate aminotransferase levels using a commercially available kit (Abbott, USA). Baseline levels were obtained from each animal at the pre-inoculation step and these values were considered references for normal values.

*Haematological and BM analysis* - The following haematological parameters were determined: haematocrit and haemoglobin levels, complete blood cell counts including reticulocytes, and total and differential leukocyte count (Horiba ABX, Japan). For evaluation of differential percentages of leucocytes and morphological analysis of BM aspirates, both samples were smeared on microscope slides and stained with May-Grünwald-Giemsa. Serum samples were also obtained by centrifugation of peripheral blood collected in tubes and stored at -20ºC for further analysis. Haematological abnormalities were analysed at the Clinical Analyses Centre of the Laboratory Animals Platform, Fiocruz.

*Liver histopathology and antigen detection* - Liver samples were fixed in 10% neutral formalin. Paraffin sections of 4 µm were stained with haematoxylin and eosin for histological study. The excised liver samples were stored at -70ºC until assayed for HAV or B19V antigen detection. Immunofluorescence for detection of HAV antigen was performed using anti-HAV IgG1 monoclonal antibody as the primary antibody (0.1 mg/mL) at 1:20 dilution (United States Biological, USA). For B19VAg detection, an anti-B19 monoclonal antibody fluid ascitic 522-G3 from a mouse was diluted 1:40 (CDC, USA). As a secondary antibody for detection of both antigens, Alexa Fluor 488-labelled chicken anti-mouse IgG (2 mg/mL) diluted 1:300 (Molecular Probe, USA) was added. Slides were counterstained with Evans Blue and coverslipped with Slow Fade in glycerol with DAPI stain (Molecular Probe) The images of positive fields were obtained using confocal microscopy with a LSM Zeiss 510 Meta (Carl Zeiss, Germany). Noninfected with HAV or B19V liver sections were used as control samples.

*HAV and B19V genome detection and quantification* - HAV RNA load was determined by RT-PCR using the TaqMan system (Applied Biosystems 7500 Real-Time PCR System, Applied Biosystems). To quantify HAV RNA, a standard curve of a recombinant clone of the 5’ noncoding (NC) region of the HAV (strain HAF-203) genome was used (27). Specific primers for HAV 5’ NC and a single labelled 5’ FAM probe were designed by our group.

To detect and quantify B19V DNA from serum of the inoculated monkeys, a real-time B19V PCR was performed using primers that were previously described for amplification of all three B19VV genotypes (Nguyen et al. 2002). Quantification was carried out using SYBR Green in the reaction mix (SYBR Green PCR Master mix; Qiagen) with a Roche Lightcycler and compared to a set of plasmid standards and to the WHO B19V Standard (National Institute for biological Standards an Control). To B19VDNA genotyping, the seminested PCR for partial amplification of the capsid gene, and the sequence analysis of the 476-bp fragment were carried out as described above.

*Statistical analysis* - The means ± standard error of the means were analysed by two-way ANOVA. The statistical analysis and graphs were performed using GraphPad Prism 5.0 software and Medcalc Software v.14.8.1 (medcalc.org/). A p-value < 0.05 was considered statistically significant.

## RESULTS

*Clinical findings* - None of the monkeys inoculated with B19V and/or HAV showed typical clinical signs of acute hepatitis or liver failure (jaundice, coagulopathy, and encephalopathy). The average rectal temperature and body weight did not change throughout the study.

**Fig. 1:**
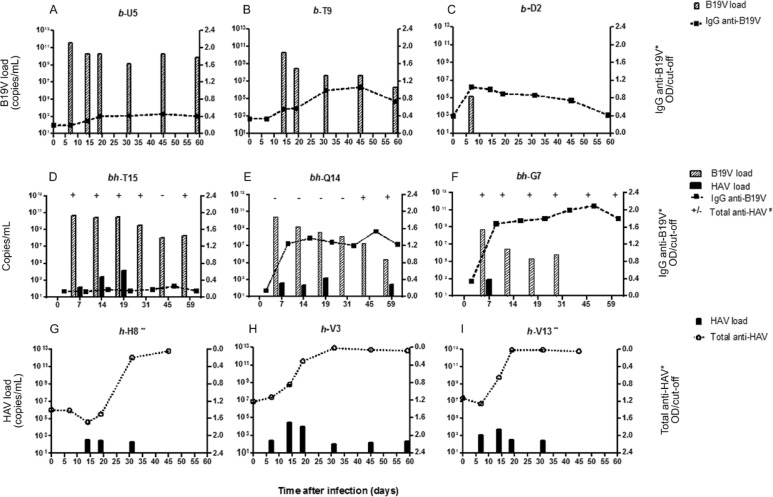
: individual analysis of the course of human parvovirus B19 (B19V) and hepatitis A infection in cynomolgus. During the time course, serum was assayed for viruses load [B19 DNA and/or hepatitis A virus (HAV) RNA] and specific antibodies (IgG anti-B19 and/or total anti-HAV antibodies). *IgG anti-B19 and total anti-HAV are shown in optical density (OD)/cut-off. IgG anti-B19 DO/cut-off ≥ 0.41 were positive. Total anti-HAV DO/cut-off ≤ 1.2 were positive; **: animals (*h*-H8 and *h-*V13) euthanised at 45 days post-infection; #: total anti-HAV detection in co-infected animals (+: positive; -: negative).

**Fig. 2:**
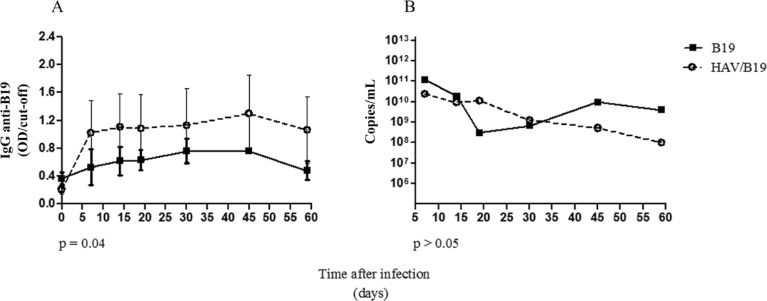
: comparative analysis of DNA load and means of antibody titres from parvovirus B19 (B19V) infected groups. A: IgG anti-B19 difference among mono and co-infected animals; B: B19 DNA detection among mono and co-infected groups. The results optical density (OD) were expressed as means ± standard error of the means. HAV: hepatitis A virus; *: p < 0.05; **: p < 0.01.

*Antibody detection* - The profile of antibodies titres are summarised in the Fig. 1. The seroconversion of the B19 groups was considered when the optical density (OD)/cut-off values were ≥ 0.41, according to ROC curve. All animals from B19 group presented IgG anti-B19 detected by EIA. An early elevation of B19 IgG levels at 7 dpi was detected in*b*-D2, while monkeys *b*-U5 and*b*-T9 showed increasing titres from 14 dpi reaching the highest titres on 45 dpi (Fig. 1). In the co-infected group, B19V IgG antibodies were detectable in *bh*-Q14 and*bh*-G7 from 7 dpi. The average of B19V antibody titres in the co-infected group was significantly higher (p = 0.04) than in the monoinfected animals (Fig. 2A). A similar increasing pattern of IgG anti-B19 antibodies titres was seen in both groups, from 14-45 dpi, followed by a gradual decreasing until the end of the study. On the other hand, the anti-B19 IgG antibody was not detectable in *bh*-T15. Total anti-HAV immunoglobulins were detected from 7-59 dpi in B19/HAV and from 14 dpi in HAV groups. Exceptions were seen in *bh*-Q14 and *h*-H8, which had a delayed detection of total anti-HAV (Fig. 1).

**Fig. 3:**
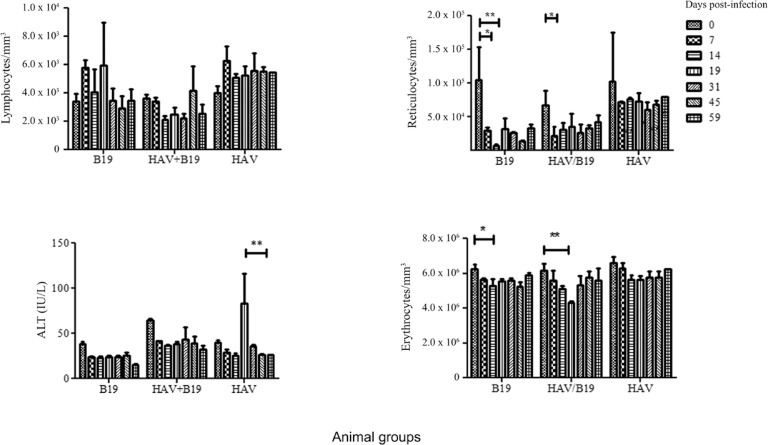
: means of haematological and biochemical values obtained from cynomolgus monkey inoculated with parvovirus B19 (B19V), B19/hepatitis A virus (HAV), and HAV. The results were expressed as means ± standard error of the means. *: p < 0.05; **: p < 0.01.

**Fig. 4:**
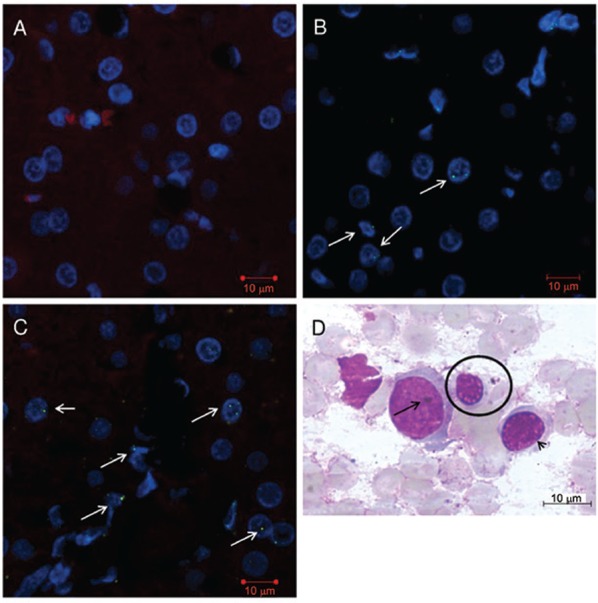
: experimental hepatitis A virus (HAV) and parvovirus B19 co-infection in cynomolgus monkey (*Macaca fascicularis*). A: absence of B19 antigen in control animal inoculated with HAV; B: nuclear and cytoplasmatic fluorescence markers to B19 antigen were detected at 59 days post-infection (dpi) in monkeys*bh*-Q14, *bh*-G7, and*bh*-T15, respectively (arrows); C: confocal scanning image. B19 antigen stained in green by indirect immunofluorescence (Alexa Fluor® 488), the liver parenchyma counterstained in bright red (Evans blue), and nucleus shown as blue (4’,6-diamidino-2-phenylindole); D: bone marrow smears from monkey *b*-D2 at 7 dpi, May-Grünwald-Giemsa stain showing three different stages of erythroid cells under light microscopy analysis. Note a giant pro-erythroblast with loose chromatin and two possible viral inclusions (arrow), a polychromatic erythroblast with more structured nucleus (arrowhead), and a smaller orthochromatic erythroblast (normoblast) with darker nucleus (circle).

*Haematological and BM analysis* - Data from blood cell counts and BM analysis were consistent with active B19V infection. In general, average reticulocyte counts showed a statistically significant reduction (p < 0.05) in both mono and co-infected groups (Fig. 3) in comparison with baseline values (pre-inoculation values). The reticulocyte counts reduction occurred just after the first or second week post-inoculation. Signs of complete recovery were not detected in these groups until the end of the follow up (Fig. 3). The average for haematocrit, red blood cell (RBC) count, and haemoglobin concentration were significantly reduced in the co-infected group at 19 dpi (p < 0.01) (Fig. 3). In the BM analysis of the B19V group, *b-*U5 showed a paucity of megakaryocytes at 31 and 45 dpi and lower BM cellularity was also found. In animal *b*-D2, an absence of megakaryocytes was detected at 19 dpi and was associated with marked decreasing of BM cellularity. The BM smear observed for monkey *b*-D2 at 7 dpi showed nuclear B19V inclusions in basophilic erythroblast (Fig. 4D). In*b*-T9, megakaryocytes were observed despite poor cellularity of BM. In the co-infected HAV and B19 group, BM aspirates revealed a strong reduction of megakaryocyte numbers associated with very poor cellularity of BM in cynomolgus*bh*-T15 and *bh*-Q14 as of 14 dpi.

**Fig. 5:**
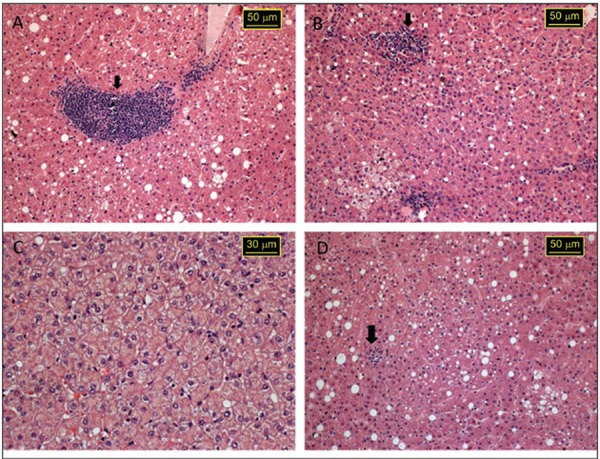
: liver histopathology of cynomolgus monkey after experimental co-infection at 59 days post-inoculation with hepatitis A virus (HAV) and parvovirus B19 stained with haematoxylin and eosin. A: moderate portal inflammation (arrow, monkey *bh*-G7); B: focus of hepatic necrosis (arrow) and steatosis (monkey *bh*-G7); C: moderate and diffuse tumefaction of hepatocytes (monkey*bh*-T15); D: numerous foci of lobular inflammation (arrow) in liver parenchyma (monkey *bh*-T15).

*Liver histopathology and blood biochemistry* - Histopathological findings were consistent with changes induced by HAV in non-human primates, which are described in the Table. Experimental co-infection with HAV and B19V demonstrated a slight worsening in liver injury during the investigated period, as moderate portal inflammation (*bh*-G7) (Fig. 5A), hepatic necrosis and steatosis (*bh*-G7) (Fig. 5B), moderate and diffuse tumefaction of hepatocytes (*bh*-T15) (Fig. 5C), and numerous foci of lobular inflammation in liver parenchyma (*bh*-T15) (Fig. 5D).

**TABLE:** Summary of comparative results from liver histological changes of cynomolgus inoculated with hepatitis A virus (HAV) and parvovirus B19 (B19V)

	HAV inoculated animals (dpi)			HAV and B19V co-inoculated animals (dpi)		
Liver lesions	H8	V3	V13	G7	Q14	T15
Microvesicular steatosis	0	1 (19)	1 (14-19)	3 (45-55)	1 (14)	1 (14)
Hepatocellular swelling	Absence	Absence	Absence	Presence (19)	Presence (14)	Presence (19-55)
Hepatocytes ballooning	Presence (19)	Absence	Absence	Absence	Absence	Absence
Intra-acinar inflammation	1 (14) 1 (31)	1 (55)	1 (19) 2 (45)	2 (55)	1 (45-55)	1 (55)
Portal inflammation	1 (19) 2 (31)	1 (19-55)	1 (19) 2 (45)	1 (19) 2 (55)	1 (45-55)	1 (31) 1 (55)
Apoptosis	0	0	0	0	1 (55)	1 (55)
Congestion	1 (14)	0	0	0	0	0
Mononuclear cells into the sinusoids^*a*^	2 (19-31)	0	0	0	0	0

*a*: type of cells present into the sinusoids; absence (0), minimal (1), moderate (2), severe (3)/days post-inoculation; dpi: days post-infection.

In this model, necroinflammatory lesions in the lobule or surrounding portal area were considered the most representative findings of viral hepatitis because these findings have never been diagnosed in liver sections during pre-inoculation analysis, including in cynomolgus inoculated with only B19V. At the interface between the portal tracts and the parenchyma, there were diffusely scattered macrophages mixed with lymphocytes and a few polymorphonuclear leucocytes. The most representative B19V antigen finding was in the nucleus and/or in the perinuclear cytoplasm of hepatocytes, which was detected in B19V inoculated animals by immunofluorescence at 59 dpi (Fig. 4A-C). Although the average of ALT level showed only a slight elevation during the course of the co-infection, the HAV-infected animals showed a significantly increase of this liver enzyme at 19 dpi (Fig. 3). Other parameter describing liver function did not present significant alterations (p > 0.05) in none of the three groups.

Molecular characterisation and VL of B19V and HAV B19VDNA recovered from serum of the inoculated monkeys (*b-*T9, *b-*U5, and*bh-*G7) showed 100% sequence identity with the original virus inoculum and were characterised as genotype 1a (GenBank accessions 34342656-KU342658). In general, B19V DNA was detected in all inoculated monkeys with the highest VL at 7 dpi (10^11^ copies/mL) followed by a gradually decreasing pattern until 59 dpi. The unique apparent B19V clearance of peripheral blood occurred in *bh*-G7 from 45 dpi. Animal *b*-D2 showed short and low viraemia, with a serum VL of 10^5^ copies/mL at 7 dpi (Fig. 1C).

The HAV inoculum was characterised as genotype 1B (Amado et al. 2010). In the HAV group, viraemia was detected from 7 dpi (*h*-V13 and *h*-V3) to 59 dpi, with the highest load detected at 14 dpi (10^4^ copies/mL) and with a gradual decrease to 10^2^ copies/mL onwards (Fig. 1G-I), while co-infected animals (*bh*-T15 and *bh*-G7) showed a short HAV viraemia until 20 dpi (Fig. 1D, E). Intermittent HAV viraemia was observed in one co-infected animal (*bh*-Q14) as it became undetectable on day 30, but reappeared on 59 dpi (Fig. 1C). A similar decreasing pattern of B19 VL was seen in both groups (B19V and HAV/B19V) until the end of the study. Comparatively, the VL of B19 group was significantly lower them that of co-infected animals (p = 0.001) (Fig. 2B).

## DISCUSSION

In this study, it was monitored the experimental infection of cynomolgus monkey (*Macaca fascicularis)* after intravenous inoculation with B19V and HAV over 59 days. All animals studied in the present work consisted of immunocompetent, clinically healthy, anti-B19V IgG negative, and the indicative signs that cynomolgus were infected with human B19V *via* the intravenous route were based on changes in haematological and virological parameters.

Our serological findings after inoculation showed a progressive IgG titre elevation (OD/cut-off rate) in almost all inoculated animals of both B19V-infected groups. The only exception was the *bh*-T15 co-infected animal, since it did not show seroconversion. This discrepant result may reflect the high viraemia (average of 10^10^ copies/mL) detected in this animal from 7 dpi onwards. Previous studies have demonstrated that in highly viraemic samples of patients with B19V infection, large amounts of IgG anti-B19V may be complexed with viral particles, becoming undetectable by serological assays. In samples with low B19V load, the specific IgG anti-B19 may be detected by ELISA, due to an excessive amount of antibody in relation to VL (Bredl et al. 2011). This picture was evident in the animal *b*-D2 which showed detectable B19V IgG titres associated with reduced viraemia only at 7 dpi (10^5^ copies/mL).

A high and persistent B19 viraemia was detected throughout the study up to 59 dpi in most of animals, even after antibody seroconversion. Similar findings were reported in human acute cases, where it is well documented that B19V clearance from peripheral blood after acute infection may last several months despite the presence of specific IgG anti-B19V (Musiani et al. 1995, Lindblom et al. 2005, Mogensen et al. 2010).

In this study, the course of infection was monitored by detectable B19V-DNA and HAV-RNA in serum samples in association with drops in RBC parameter, particularly reticulocytopenia, mild to moderate anaemia, as well as detection of a nuclear inclusion in basophilic pro-erythroblast and B19V Ag detection in liver samples during the 59th day of investigation. These results were also reported by other authors in natural Simian parvovirus (SPV) infection and in experimental B19V infection using human beings as volunteers (Anderson et al. 1985). In our understanding, the B19V tropism for erythroid progenitor cells induced a red cell depression during the first 10 days of experimental infection, as previously described in humans infected with B19V by others (Anderson et al. 1985, Potter et al. 1987). These findings were not detected in HAV-inoculated animals.

Despite our short-term study, our data suggest that cynomolgus monkey developed a benign pattern of B19V infection similar to human disease, which was characterised by a higher and persistent viraemia associated with antibody detection and erythroid precursor’s depression (Potter et al. 1987,Bredl et al. 2011). The presence of B19V infection was associated with moderate laboratory findings of reticulocytopenia, a decrease in erythrocyte counts and haemoglobin content, marrow hypocellularity, and a reduced number of megakaryocytes, with a less pronounced effect on the other hematopoietic cells. A severe haematological disorder, induced by SPV, has been previously reported only in immunocompromised cynomolgus (Green et al. 2000, Schröder et al. 2006).

The finding of erythroid lineage depression, which was the most common laboratory feature described in human B19V infection, was confirmed in our study in B19V-infected cynomolgus. This transitory hypoplasia has been explained by B19V NS1-induced apoptosis in erythroid lineage (Moffatt et al. 1998, Sol et al. 1999,Yaegashi et al. 1999). Interestingly, cynomolgus inoculated with only B19V showed a more pronounced reduction in reticulocyte counts during the first 15 dpi, while in the co-infected group this finding was observed earlier at the first week post-inoculation. Haematological complications in human beings have also been reported in HAV infection (Aoyagi et al. 1987, Chatzimichael et al. 2008, Rauff et al. 2011), but have never been described in immunocompetent cynomolgus. In our study, erythroid hypoplasia was more evident in the co-infected group, which may be also explained by a synergic effect of both infections or overproduction of interferon-gamma and tumour necrosis factor-alpha in the course of co-infection, as described by others (Morceau et al. 2009).

None of the HAV inoculated monkeys exhibited jaundice related to hepatitis infection, as has been reported by our group (Pinto et al. 2002, Amado et al. 2010). However, an increased ALT level, viral shedding, and histopathological findings of B19/HAV and HAV infections were consistent with changes induced by HAV in New World non-human primates (Pinto et al. 2002). As observed by other authors, B19V antigen could be detected in liver parenchyma cells of B19V inoculated monkeys.

However, the B19V and HAV co-infection did not induce an ALF in the co-infected cynomolgus monkeys. The mechanism by which B19V causes hepatic injury remains unclear, but it has been proposed the hepatic cell damage as a consequence of a direct invasion by the virus (Poole et al. 2004). A possible explanation is the presence of the globoside, the main cell receptor for the virus that can be detected on the membranes of the hepatocytes (Koliou et al. 2014). It seems that despite the inability of the virus to replicate in a nonpermissive cell, as hepatocytes, it retains its ability to produce the nonstructural protein NS1, which induces arrest at G1 phase. The G1 arrested hepatocytes undergo apoptosis by activation of caspase-3 and caspase-9, supporting the hypothesis of the direct cytopathic effect of B19V on liver cells (Poole et al. 2004). Additional studies concerning molecular mechanisms of liver injury during HAV and B19V co-infection should be theme for further experimental infections. Regarding HAV-induced hepatic injury, it has been proposed an immune mediated mechanism triggered by the HAV infection. Therefore, the absence of signals of liver failure in our study may reinforce the role of individual immune response in ALF as also observed in human host (Jeong & Lee 2010).

In summary, our observations showed that hepatitis due to co-infection HAV and B19V do not seem to cause severe acute hepatic illness in the cynomolgus model. These findings are in accordance with previous studies, which demonstrated that multiple hepatotropic virus co-infections in humans might not result in more severe acute hepatitis (Kumar et al. 2006, Wang & Zhang 2009). This preliminary study also contributes with a new finding, that the in vivo demonstration of susceptibility of cynomolgus to human B19V qualifies this animal as a useful model for B19V pathogenesis studies.
